# Cross-sectoral and cross-agency team science: successful strategies from the National Center for Advancing Translational Sciences

**DOI:** 10.3389/fpsyg.2026.1790707

**Published:** 2026-05-19

**Authors:** Mark F. Miller, Ann R. Knebel, Sharie J. Haugabook, Elizabeth A. Ottinger, David M. Reif, Amanda L. Vogel

**Affiliations:** 1 National Center for the Advancement of Translational Sciences, National Institutes of Health, Rockville, MD, United States; 2 National Institute of Environmental Health Sciences, National Institutes of Health, Durham, NC, United States

**Keywords:** cross-disciplinary research, drug development, team science, toxicology, translational science, project management

## Abstract

The National Center for Advancing Translational Sciences (NCATS) intramural Division of Preclinical Innovation (DPI) has employed since its inception a team-based scientific research model that emphasizes interdisciplinary and multidisciplinary collaborations to advance its mission to develop biomedical treatments for patients faster. The team-based scientific approach creates a collaborative research environment and has been credited with driving NCATS DPI success. This article analyzed the organizational policies, structures and processes that support the team science environment at DPI. Two case studies are presented to highlight implementation strategies and broadly applicable key principles that have enabled team science success. These key principles include: Coordination of Operations, Stakeholder Engagement and Communication, Risk Management and Strategic Decision-Making, Progress Assessment and Resource Monitoring, Alignment of Program Expectations, Nimble Cross-partner Approaches, the Ability to Expand or Redefine Program/Project Scope, and Embracing Innovation. Although presented in the context of these two cases, the key principles are broadly applicable across a wide range of collaborations, and team-based science initiatives more generally. By sharing these examples of team science in practice, and the key principles that support success, NCATS aims to support theoretical developments and innovation in the science of team science and translational research.

## 1 Introduction

The National Center for Advancing Translational Sciences (NCATS), a part of the National Institutes of Health (NIH), focuses on developing health treatments, aiming to deliver therapies to patients faster by creating tools, technologies, and strategies for drug discovery, rare disease research and therapeutic development, and predictive toxicology. It supports innovative research and education to accelerate the process of turning scientific discoveries into diagnostics, treatments, and other health solutions that improve the health of individuals and communities (Austin, 2018). Rapid advances are often achieved by focusing on similarities across many diseases at once, rather than focusing on a single condition.

A key principle of translational science and accelerating human health discoveries is to align the capabilities of multiple stakeholders, across various disciplines, toward agreed upon scientific goals. NCATS intramural Division of Preclinical Innovation (DPI) employs a team-based scientific research model that emphasizes collaborative interdisciplinary and multidisciplinary approaches, rather than a traditional investigator-initiated approach. DPI enables effective Team Science through a combination of policies, structures, and processes that establish unified expectations and incentivize non-hierarchical, project-based, cross-disciplinary team science for maximum scientific impact. Effective DPI Team Science relies on collaborative leadership demonstrated by all members of a collaboration, particularly when executing larger, more complex, multidisciplinary projects and programs (Vogel et al., 2021; Bennett et al., 2018).

DPI continues to employ these principles of Team Science to engage extensive collaborations across NIH Institutes and Centers, academia, and industry (see Table 1), thereby assembling unique project and program teams with extensive capabilities. These capabilities typically include a combination of systems biology, chemical synthesis and optimization, new approach methodologies, automation and screening, data analytics and informatics, machine learning/artificial intelligence, drug development, technology transfer and public–private partnerships, among others. Many DPI collaborations are designed to be short-term, nimble relationships targeted to a single project, program, or outcome and leverage a subset of these capabilities, others extend long-term and are designed with inherent plasticity that allows evolution as the science unfolds and new opportunities emerge.

**Table 1:** Types of collaborations in internal research programs: comparing NCATS to the average of four other NIH Institutes and Centers with similarly sized budgets.^a^

Year	2016		2017		2018		2019		2020	
	NCATS	NIH AVG.*	NCATS	NIH AVG.*	NCATS	NIH AVG.*	NCATS	NIH AVG.*	NCATS	NIH AVG.*
Total projects	175	89.3	182	68.2	184	83	178	84.5	167	87.8
Internal projects	23	18.3	21	13.8	16	17.5	19	19.75	22	22.8
NIH collaborator only	32	9.8	27	7	34	9.5	29	9	23	9
External collaborator only	94	28	107	21.2	97	24	93	23.5	84	25.5
NIH and external collaborators	26	33.3	27	26.2	37	32	37	32.25	38	30.5
Year	2021		2022		2023		2024		2025	
	NCATS	NIH AVG.*	NCATS	NIH AVG.*	NCATS	NIH AVG.*	NCATS	NIH AVG.*	NCATS	NIH AVG.*
Total projects	146	88.25	148	90	129	87.75	149	86.25	176	84.25
Internal projects	11	22.5	20	21	17	8.75	38	52.5	42	49.75
NIH collaborator only	21	8.75	30	13.5	22	21.75	17	8.25	18	6.25
External collaborator only	78	25.25	62	20.5	49	4	34	21.75	39	22.25
NIH and external collaborators	36	31.75	36	35	41	53.25	60	3.75	77	6

a Adapted and updated from Vogel et al. (2021).

*Average across four NIH Institutes and Centers with budgets of similar size to NCATS.

Given DPI’s long history of accelerating innovations through team science, this article highlights two case studies that describe the strategies and approaches DPI has used to decrease barriers in translating preclinical findings to clinical application. It outlines the complexities and challenges associated with sustaining collaborations and innovating in a team science environment. One case describes the ways in which the DPI Therapeutic Development Branch (TDB) leverages partnerships to bring new treatments to patients, especially those with rare diseases; the other describes the strategies used by the Toxicology in the 21st Century (Tox21) program. These programs have addressed the challenges associated with creating and sustaining a novel Team Science research model over the past 15 years. They highlight replicable Team Science strategies that have been used successfully in partnerships across government, academia, industry, and small business.

## 2 Therapeutic drug development pipeline: cross-sectoral team science

Team science is at the core of the NCATS everyday operational model, with the entire research portfolio consisting of milestones-driven collaborations with both internal and external partners that bring complementary expertise to biomedical innovation. The TDB within the intramural DPI of NCATS plays a critical role in the drug development process, conducting comprehensive preclinical and translational research activities necessary to support preparation of an Investigational New Drug (IND) application for review by the Food and Drug Administration (FDA) before clinical trial initiation (Ottinger et al., 2014). Through programs such as Therapeutics for Rare and Neglected Diseases (TRND), TDB provides in-kind scientific, operational, and regulatory support for IND-enabling projects with NIH, academic, non-profit, and private-sector collaborators working on novel treatments for diverse diseases and conditions. Projects are strategically prioritized to address common translational roadblocks either in developing novel/emerging therapeutic compounds and/or in targeting multiple patient populations due to shared molecular mechanisms. Project proposals are evaluated thoroughly by assessments of scientific validity including strengths and weaknesses, feasibility for clinical and commercial development as well as the plans for human trials. It is critical that prospective collaborators engage with clinicians and the patient community. The TDB aims for collaborations with the highest translational impact and social value so this ensures that the proposal is of highest quality, likely to meet its stated goals and objectives and has the potential of advancing knowledge in the field. When all else is equal in the scientific evaluation criteria, and since TDB’s resources are limited, the program will then prioritize therapeutic development for diseases with no treatment under investigation or that lack preliminary data to attract the necessary funding and resources. Notably, since its inception in 2010, the TDB has provided support for over 100 collaborations, enabling >50 INDs and approval of four new drugs, after hand-off to a commercial partner (National Center for Advancing Translational Sciences (NCATS), 2026).

The therapeutic development process is complex, with many interdependent activities that are both time and resource intensive. It requires the rigorous validation of efficacy for the candidate molecule in preclinical disease models; characterizing metabolic, pharmacologic, and safety properties; developing efficient synthetic processes to manufacture the candidate drug substance and formulate the drug product for human use at sufficient scale—all while adhering to rigorous regulatory standards set by the FDA. Projects adopted into the TDB pipeline are at a phase in the translational research pipeline where critical pre-clinical data are needed to advance the drug candidates through the perceived “valley of death”—the often challenging transition period from preclinical research to a first in human clinical trial. To meet the scientific and operational challenges of advancing therapeutic development projects, a “Team of Teams” multi-team system approach is implemented. We then work closely with public and private partners to contribute the needed resources and expertise to increase the probability of regulatory success, thus lowering the financial risk and increasing commercial viability of the treatment for the benefit of the patient community.

**Figure 1:**
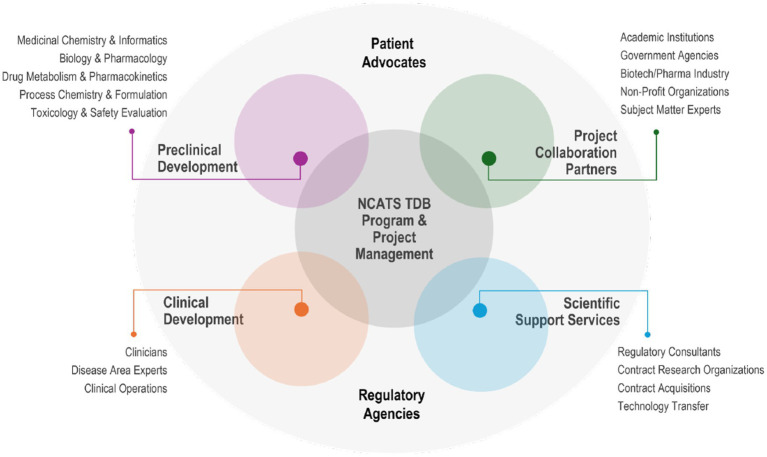
The “Team of Teams” multi-team system approach to collaboratively conduct therapeutic drug development efforts in the Therapeutic Development Branch at NCATS/DPI.

Therapeutic development teams are large and multidisciplinary, requiring diligent and continual coordination so all collaborators and activities are integrated and aligned toward common scientific and clinical objectives (Figure 1). Within NCATS, the TDB team comprises many different individuals with diverse scientific expertise—computational and medicinal chemists, molecular and cellular biologists, bioinformaticians, pharmacologists, pharmacokinetics and drug metabolism (DMPK) scientists, toxicologists, and drug manufacturing and formulation experts. Equally critical is project management. Project managers (PMs) work closely with the strategic alliance or tech transfer office, contracting technical support staff and internal/external project collaborators to execute agreements and pursue project goals and milestones. If the capability is not available in-house, it is outsourced through contracts. External partners can include disease experts (as NCATS is disease agnostic), such as scientists and/or clinicians and patient advocates, regulatory science providers, and contract research organizations. This broad range of stakeholders is essential to facilitate the development of new therapeutic interventions for societal benefit and use.

The discipline of scientific project management is strategically embedded in the TDB team science ecosystem for preclinical drug development to ensure efficient execution and achievement of milestones. Using their scientific/technical background the TDB PMs advance projects by providing consistent oversight and guidance, removing roadblocks and managing the project lifecycle, from initiation and planning through execution and closeout. Each project team includes a Lead PM, who is supported by an analyst. The Lead PMs have PhD level scientific background and training, as their role is to bring clarity, transparency, and a full understanding of the interconnections across complex technical disciplines and among the multiple partners contributing to that project. Good project management supports the execution of good science. Key objectives of an integrated project management team include:

### 2.1 Coordination of operations

PMs provide day-to-day coordination and monitoring of the therapeutic development activities, ensuring efficient planning, implementation, and execution of studies. A centralized internal PM guidebook has been created to standardize management across programs, projects, and personnel. Regular, effective, and skillfully managed communications are vital, and can mean the difference between the success or failure of a project. A centralized collaboration platform that organizes communication and document management, ex. Microsoft (MS) Teams, is used to confidentially share ideas, documents and other deliverables, and interact with other team members, within and outside of the NIH, throughout the lifecycle of the project. Each project has its own collaboration site with channels organized around the activities of each discipline for effective and timely decision-making.

### 2.2 Stakeholder engagement and communication

PMs manage fluid communication amongst the project team and other relevant partners. They manage the dedicated team collaboration space that promotes effective and efficient engagement between team members to ensure transparency and accountability. Recurring team meetings are scheduled to report on progress, review data, and discuss any emerging issues or concerns. Minutes and action items are captured for follow-up and archived. This timely exchange of information and seamless data delivery drives better decisions.

### 2.3 Risk management and strategic decision-making

PMs ensure that rising issues are addressed and resolved effectively and efficiently, with the goal of mitigating downstream disruptions to achieve milestones. Relevant information and action items are communicated promptly to the team, and PMs conduct real time risk assessment to identify early, any potential scientific or administrative issues. PMs facilitate discussions to guide the team to proactively make strategic decisions and take action to address challenges.

### 2.4 Progress assessment and resource monitoring

PMs continually assess the holistic progress of the project toward achieving milestones while managing resources and coordinating timelines. As described, the TDB operates as a “Team of Teams” multi-team system, and the respective project team includes distinct areas of scientific expertise, internally and externally, with defined roles and responsibilities focused on progress toward completion of their individual but linked objectives to meet the overall goal of each project. Each discipline sub-team is responsible for conducting specific activities and studies (e.g., toxicology, formulation development) needed to generate the required data to support an IND application and enable clinical trials. PMs work collaboratively within the team science ecosystem to foster team accountability for timelines and resourcing, and the team adapts accordingly to feedback received throughout the project lifecycle. Ultimately, by maintaining a high level of oversight and coordination, PMs ensure that all project activities within disciplines are aligned and linked to the overall goal and objectives of the therapeutic development project.

## 3 Toxicology in the 21st century: intra-governmental team science

In response to the National Research Council report on *Toxicity Testing in the 21st Century: A Vision and a Strategy* (Gibb, 2008) and to adapt toxicity testing paradigms to meet evolving regulatory needs, the National Toxicology Program [i.e., the National Institute of Environmental Health Science (NIEHS), Centers for Disease Control (CDC), and the Food and Drug Administration (FDA)], the Environmental Protection Agency (EPA), and the National Institutes of Health Chemical Genomics Center (NCGC), which would later become part of NCATS, established a novel approach to use high-throughput and computational approaches as predictors of animal toxicity studies (Collins et al., 2008). This team-based research partnership, dubbed the Toxicology in the 21st Century (Tox21) consortium, accelerated the evolution of regulatory toxicology by developing and validating new approach methods (NAMs) to rapidly and efficiently evaluate biological activities and potential human health hazards of commercial chemicals, pesticides, food additives/contaminants, medical products and environmental exposures. The vision was to shift toxicology toward higher throughput, more predictive, mechanism-informed approaches.

The Tox21 consortium, when originally established, was created in response to a core problem: traditional animal-based and low-throughput toxicology could not keep pace with the expanding universe of industrial and environmental chemicals, pesticides, food constituents, and medical products (Kavlock et al., 2009). Thus, Tox21 was intentionally designed around complementary agency strengths with shared leadership and designated areas of responsibilities: NCATS provided high-throughput screening (HTS) infrastructure and assay development capabilities; NIEHS/NTP brought toxicology expertise, *in vivo* anchoring, and translational context; EPA contributed computational toxicology and chemical prioritization resources; and FDA brought domain expertise for pharmaceuticals and food-related substances.

This collaborative architecture produced several early, concrete accomplishments that established credibility and momentum. (1) Rapid, scalable experimental capacity: By the end of Phase I, Tox21 had tested 2,800 compounds in more than 50 assays using NCATS high-throughput automated robotic screening system, demonstrating feasibility of federal-scale HTS for toxicity-relevant pathways. (2) Public-data-by-default norms: Phase I outputs were released into public databases (e.g., PubChem) and partner resources, which accelerated external method development and validation while reinforcing transparency as a program norm. (3) A common, curated chemical library and shared testing strategy: In Phase II, the program created a collaborative chemistry and screening enterprise built around a broadly relevant compound collection (the “10 K library”) to enable systematic comparisons across assays, targets, and agencies. (4) A recognizable “platform” for national toxicology modernization: The consortium’s early progress (including the start of the 10 K library using high-speed robotics) helped establish Tox21 as a practical engine for 21st-century toxicology rather than a purely conceptual initiative (Tice et al., 2013).

Having demonstrated the proof-of-concept goals regarding HTS, and producing the largest set of publicly available toxicological dose response data ever assembled, the consortium had matured into an operationally robust, data-centric enterprise. In 2018, Tox21 leadership revisited the strategic plan in an effort to explicitly broaden the scope of the collaboration beyond initial high-throughput assay panels and early-phase screening (Thomas et al., 2018). The new strategic approach expanded testing to more compounds and implemented a fully automated platform capable of profiling the full collection at scale. This evolution also reflected a shift from “screening as an end” to “screening as a launchpad” for integrated evidence generation across multi-omics platforms. Key features underpinning the maturation of Tox21 include stronger informatics and artifact-control practices, reporducible screening at consortium scale, and a shared data ecosystem (Huang, 2022).

The 2018 Tox21 strategic plan (Thomas et al., 2018) further established a novel programmatic mechanism for cross-partner projects among two or more agencies, leveraging resources toward entirely new research directions. This approach allowed alignment of government agency missions, cross-disciplinary and trans-disciplinary team science for complex toxicology challenges. These experiences and flexibility allowed establishment of a novel operational structure, which fostered its evolution, and provided insights into the reasons why the Tox21 consortium has been successful through changing research environments and shifts in organizational priorities.

### 3.1 Aligning expectations at the program/project level

Tox21 continues to serve as a model for team-based scientific collaboration because it is not a “shared workload” model; it is a shared-value model. Each agency’s incentives are explicit and legitimate (e.g., NCATS technology development, assay performance, and translational drug discovery; NIEHS/NTP translational toxicology; EPA chemical prioritization and computational integration; FDA product-safety context). The program’s goals for mechanism discovery, prioritization, and improved predictive models are broad enough to accommodate different agency missions while still producing a coherent joint deliverable of actionable, publicly reusable datasets.

### 3.2 Nimble cross-partner team science

By design, each partner brings distinct value/utility, and not all partners are required on every project. The consortium uses a hub-and-spoke logic: core infrastructure and standards remain stable, while project teams assemble based on the scientific question. This lowers coordination cost and allows mobilization of the right expertise (e.g., assay engineering, computational modeling, or domain biology) without requiring every agency to be equally engaged in every activity. Cross-partner projects formalize this nimbleness by providing an entry point for discrete, time-bounded, question-driven collaborations.

### 3.3 Built-in capacity to expand program/project scope using new methodologies

Tox21’s strategic evolution explicitly supports incorporation of new data modalities. The consortium is no longer defined by receptor and stress-response reporter assays. It is positioned as a framework to integrate emerging new approach methodologies (NAMs), high-throughput transcriptomics, and other data sources, into toxicity pathway development and evidence integration. This expansion is critical for keeping the platform scientifically contemporary and for enabling richer mechanistic anchoring of screening results.

### 3.4 Embracing innovation and new partnerships

Tox21 has maintained relevance by enabling investigator-initiated innovation (via cross-partner projects) rather than constraining work to a fixed assay catalog. For example, NIEHS and NCATS in collaboration with academic research hospitals used information from large-scale screening of the Tox21 10 K library to support studies in human-relevant neuroendocrine models to identify chemicals potentially linked to early puberty (Yang et al., 2024). This illustrates how the platform can catalyze new, cross-disciplinary partnerships and translate screening signals into mechanistic biology and public-health-relevant hypotheses.

## 4 Discussion

The National Academies of Sciences, Engineering, and Medicine recently published a consensus study report on the science and practice of team science (National Academies of Sciences, Engineering, and Medicine (NASEM), 2025), highlighting the importance of interdisciplinary team-based approaches for complex scientific investigation and the opportunities and challenges of implementing team science in the currently evolving research environment. The report explored collaboration practices across a wide range of scientific disciplines, team sizes, program complexities, modalities of engagement, and other characteristics which have proven critical when assessing implementation approaches. Finally, although the NASEM report aimed to identify evidence-based best practices for assembling and managing scientific teams, they also recognized a disconnect between theory, empirical evidence, and accepted best practices for implementing team science initiatives.

As a collaborative, team science organization, NCATS has a wealth of real-world, practical experience building highly diverse project and program teams to advance its vision to deliver more biomedical treatments, to all people, more quickly by leveraging the NCATS Translational Science Principles (Faupel-Badger et al., 2022). NCATS team science collaborations utilize principled frameworks for multidisciplinary research by focusing on shared goals and leadership, clear roles, effective project management, transparent communication, and building mutual trust in other’s abilities, honesty, reliability, and intentions. While no project is without challenges requiring problem solving, strong collaborative relationships and team identity have provided the foundation for working through critical differences in perspective.

By building upon replicable team science strategies, NCATS has successfully partnered across government, academia, industry, and small business. The cases presented describe highly specialized team science applications that may not be broadly applicable, but they highlight best practices and key features that have proven useful across the entire NCATS collaborative research portfolio. Each case study describes a unique collaborative approach. The therapeutic drug development pipeline example details a specific approach for partnering with private industry to advance drug candidates to FDA Investigational New Drug submission. This approach is applicable for various drug candidates across multiple independent research partnerships. The Tox21 example on the other hand demonstrates the evolution of a single research consortium to expand the scientific collaboration space and redefine the operating model to better support the consortium’s shared goals.

The key principles highlighted for each case study are applicable across NCATS partnerships. For example the principles of Coordination of Operations, Stakeholder Engagement and Communication, Risk Management and Strategic Decision-Making, and Progress Assessment and Resource Monitoring identified in the TDB example are equally applicable in the Tox21 consortium context. Similarly, therapeutic drug development team science requires the principles of Alignment of Program Expectations, Nimble cross-partner approaches, the Ability to Expand or Redefine Scope, and Embracing Innovation, identified in the Tox21 case.

The science of team science requires the identification of specific use cases that demonstrate successful team science implementation so the findings can be used to validate generalizable best practices and identify the requirements for institutional support. By sharing these examples of team science in practice, and the key principles that allowed them to be successful, NCATS aims to support theoretical developments and innovation in the science of team science and translational research more generally.

## Data Availability

The original contributions presented in the study are included in the article, further inquiries can be directed to the corresponding author.
